# A Biomimetic Platelet-Rich Plasma-Based Interpenetrating Network Printable Hydrogel for Bone Regeneration

**DOI:** 10.3389/fbioe.2022.887454

**Published:** 2022-04-12

**Authors:** Shijia Tang, Lin Wang, Yunyang Zhang, Feimin Zhang

**Affiliations:** ^1^ Jiangsu Province Key Laboratory of Oral Diseases, Department of Prosthodontics, The Affiliated Stomatological Hospital of Nanjing Medical University, Nanjing, China; ^2^ Center of Modem Analysis, Nanjing University, Nanjing, China

**Keywords:** platelet-rich plasma, interpenetrating polymeric network hydrogel, osteogenic differentiation, bone regeneration, biomineralization

## Abstract

Repair of bone defects caused by trauma or diseases is the primary focus of prosthodontics. Hydrogels are among the most promising candidates for bone tissue regeneration due to their unique features such as excellent biocompatibility, similarities to biological tissues, and plasticity. Herein, we developed a type of novel biomimetic interpenetrating polymeric network (IPN) hydrogel by combining methacrylated alginate and 4-arm poly (ethylene glycol)-acrylate (4A-PEGAcr) through photo-crosslinking. Platelet-rich plasma (PRP), a patient-specific source of autologous growth factors, was incorporated into the hydrogel, and thereafter the hydrogels were biological mineralized by simulated body fluid (SBF). Physical properties of hydrogels were comprehensively characterized. *In vitro* studies demonstrated that the incorporation of PRP and biomineralization promoted the biocompatibility of hydrogel. Strikingly, the osteogenic bioactivities, including ALP activity, mineralized nodule formation, and expression of osteogenic markers were found substantially enhanced by this biomineralized PRP-hydrogel. Finally, a rabbit model of bone defect was employed to assess *in vivo* bone regeneration, micro-CT analysis showed that the biomineralized PRP-hydrogels could significantly accelerate bone generation. We believed that this novel biomineralized PRP-incorporated IPN hydrogel could be promising scaffolds for bone tissue regeneration.

## Introduction

Repair of bone defects is the primary focus of prosthodontics, especially with an increasing demand for bone grafts to heal bone defects due to trauma or diseases (Schneider et al., 2009). Although autografts and allografts remain commonly used clinically for the reparation of large bone defects, such approaches still have some drawbacks. For example, autografts could cause a secondary injury when obtaining donor tissue, while allografts pose a potential risk of pathogen transmission and immune rejection (Fleming et al., 2000; Lei et al., 2019; Busch et al., 2021). With the development of biotechnology, bone tissue engineering (BTE), which is expected to overcome these shortcomings, might provide a new tool for the treatment of bone defects (Zheng et al., 2022). The next-generation engineered bone tissues aim at mimicking physiological tissue morphology and functions by generating more complex and structurally organized implants (Hofmann et al., 2007). The scaffold is one of the critical components in BTE. It acts as a temporary substrate or template as well as a carrier of biochemical factors, which can provide cells with both anchorage sites and appropriate physical (e.g., mechanical properties) and biochemical stimulation (e.g., cytokine, chemokine), therefore supporting cell growth and maintaining cell functions. In particular, the architecture of scaffold provides spatially mechanical stimulation to cells and defines the final shape of the newly formed bone (Schmid et al., 2019). Therefore, the outcome of bone tissue regeneration strategies is dependent, to a large extent, on the performance of scaffolds (Wang et al., 2007; Seok et al., 2021).

There are several kinds of biomaterials, including hydrogels, biological ceramics and collagen that have been developed as promising scaffolds for BTE (Huang et al., 2021; Li et al., 2021; Wang et al., 2021). Among them, hydrogels possess highly hydrated polymer networks, numerous functions towards cells and have been extensively utilized as bone substitute in the field of tissue engineering (Rastogi and Kandasubramanian 2019). Hydrogels are also good space filling agents (flexibility in fitting in any application site), delivery vehicles for bioactive molecules (controllability pore size in the polymer network), and three-dimensional culture matrices (Drury and Mooney 2003; Tan et al., 2005; Ma et al., 2020). All these advantages make hydrogels a promising candidate for using in bone tissue engineering scaffolds. Recently, interpenetrating network (IPN) hydrogels with two or more networks have attracted considerable attention in the field of BTE due to the enhanced mechanical strength and toughness (Scalet et al., 2021; Zou et al., 2021). There are several studies that have demonstrated that the mechanical performance of IPN hydrogels was far superior compared with either of the “parent” networks (Ingavle et al., 2016). However, the application of IPN hydrogels is still limited since the inability to provide cells with proper microenvironment, such as lack of cell adhesion sites and therefore unable to induce bone regeneration. Therefore, it is highly desirable and a great challenge to prepare IPN hydrogels-based scaffolds with excellent biological activities for bone tissue regeneration.

We hypothesized this lack of cell compatibility can be substantially improved by combining synthetic IPN hydrogels and bioactive agents, including nanocrystalline hydroxyapatite (nHAp), calcium phosphate and growth factors (Li C. et al., 2020; Dabiri et al., 2021). Among them, growth factors are essential for successful bone regeneration and their importance has been shown in the previous study (Boyle et al., 2013; Wu et al., 2020). Platelet-rich plasma (PRP) is a mixture of highly concentrated platelets and associated growth factors, including platelet derived growth factor (PDGF), vascular endothelial growth factor (VEGF), transforming growth factor-β (TGF-β), fibroblast growth factor (FGF) and insulin-like growth factors I (IGF-I) (Foster et al., 2009; Franchini et al., 2018). Numerous studies have shown the effectiveness and versatility of PRP in regeneration/repair of skin, cartilage and bone (Hom 1995; Rodriguez et al., 2013). Particularly in the case of bone regeneration, PRP-based scaffolds were demonstrated to be capable of enhancing bone density/mineralization, vascularization and osteogenesis (Rai et al., 2007; Zhou et al., 2021).

In the present study, we fabricated a novel biomimetic PRP-incorporated methacrylated alginate/4Arm-PEGAcr IPN hydrogels through photo-crosslinking upon exposure to long-wave UV light and further biological mineralized through exposure to native calcium ions in simulated body fluid (SBF). Cell proliferation and adhesion were measured to investigate their biological activities using BMSCs. After that, Alkaline phosphatase (ALP) activity level, Alizarin red staining, expression of osteogenic-related genes and proteins were detected to investigate their osteogenic bioactivities. Moreover, the IPN hydrogels were surgically implanted to a rabbit condyle defects for *in vivo* bone regeneration assessment. We believe that the biomineralized PRP-incorporated IPN hydrogel can be used as promising scaffolds for bone tissue regeneration.

## Materials and Methods

### Materials

Rabbit bone marrow mesenchymal sem cells were purchased from Cyagen Co. Ltd. (GuangZhou, China). Sodium alginate, methacrylic anhydride, 2-hydroxy4′-(2-hydroxyethoxy)-2-methylpropiophenone (Irgacure 2959, 98%) and sodium hydroxide (NaOH) were purchased from Sigma-Aldrich Co. Ltd. (MO, United States). 4-arm poly (ethylene glycol) acrylate was provided by Sinopeg Biotech Co. Ltd. (China). Ultrapure water was obtained from a Millipore Auto-pure system. All regents were used without further purification.

### Synthesis of Methacrylated Alginate

The methacrylated alginate was prepared according to a reported study (Choi et al., 2021). Briefly, ALG was dissolved with 20 ml of deionized water to produce a 2% (w/v) solution at 37°C overnight. Then, 20 ml of methacrylic anhydride was added dropwise to the system, and the solution was continuously stirred magnetically at room temperature for 3 days, with the pH periodically adjusted to 7 with aqueous NaOH (5 M). After the 3-days reaction period, the resulting solution was poured into 100 ml of ethanol (pre-chilled in advance at -20°C) to precipitate the ALG-MA product for about 8 h at RT. Finally, the precipitate was vacuum filtered, washed three times with ethanol, oven dried at 37°C, and stored at −20°C until use. From the addition of methacrylic anhydride, all the synthesis steps were carried out under dark conditions.

### PRP Preparation

The PRP was prepared according to a reported study (Faramarzi et al., 2018). Briefly, rabbit ear margin vein blood was centrifuged at 250 g for 15 min to separate red blood cells from plasma. The upper plasma phase, including the interface, was centrifuged at 1600 g for 60 min to pellet the platelets. The upper three-quarters of the plasma phase were discarded to retain the remaining one-quarter. The obtained PRP was then stored at -80°C until further use. Further platelet activation was performed by repeated freeze-thaw cycles in subsequent experiments.

### Preparation and Structural Characterization of Hydrogels

To generate hydrogels, alginate-methacrylic anhydride was dissolved separately in PBS (PRP group: 10% PRP [v/v]-ALGMA) to achieve a concentration of 1.67% (w/v) in borosilicate vials, combined with four arm-PEG at twice the solution’s mass, and incubated in a 37°C water bath until complete dissolution. The resulting solutions were added with I2959 (1% (w/v)). Then, 180 µL of the resulting solution was solidified in PDMS molds (diameter, 1.3 mm; height, 1 mm) to form sheets and subjected to UV-light irradiation for 90 s at 365 nm. Finally, these hydrogel disks were soaked in dopamine (2 mg/ml) and collagen for 60 s after being submerged in PBS or SBF for 24 h to remove the excess unreacted monomer. For *in vitro* experiments, all hydrogel disks were sterilized by ethylene oxide.

#### Nuclear Magnetic Resonance (1H NMR) Spectroscopy

The samples were examined before and after the ALG-MA grafting reaction on a solid-state NMR spectrometer, and 1H NMR spectra were obtained and analyzed for comparison. Magnetic field intensity was 9.4 T, with a maximum speed of 10 kHz.

#### Fourier Transform Infrared Spectroscopy

The material was dried in the oven, mixed with potassium bromide (KBr) powder and ground into a transparent flake with a mass ratio of 1:100 of KBr powder. Spectral analysis of the sample was performed by FTIR, at a resolution of 4 cm^−1^ and an instrument scan range of 400–4,000 cm^−1^.

#### Swelling Ratio Measurement

PBS-PRP (-), PBS-PRP (+), SBF-PRP (-) and SBF-PRP (+) hydrogels were fabricated and lyophilized. After measuring the lyophilized weight, the lyophilized hydrogels were immersed in PBS or SBF at room temperature, removed at certain time intervals, and weighed again after wiping off the surface water with filter papers until they were completely swollen and the weight would not increase further. The swelling ratio was calculated by the following equation: swelling ratio (Q) = W_s_/W_l_, where W_s_ is the weight of a fully swollen sample and W_l_ is the weight of the corresponding lyophilized sample. Four replicates were used in this experiment.

#### Mechanical Test

For compressive mechanical testing, hydrogels were produced in a cylindrical shape (hight: 10 mm, diameter: 10 mm). The compressive test was performed on a Trapezium X-type tester (Shimadzu Corporation, Japan). The horizontal head of the mechanical testing machine was set to move at a speed of 1 mm/min. The end of the sample was fixed with a metal clamp, and then a force gradient of 0.25 N/min was applied, starting from 2 mN preload until the sample fractured. The stress-strain curve was generated for each sample, and the maximum fracture point was recorded to calculate the strain results. The Young’ modulus could be obtained by calculating the slope of stress-strain curve in their initial linear part. The energy dissipation of hydrogels was obtained by calculating the area of compression-relaxation cycles. Triplicate assays were performed thrice. The rheology properties of the hydrogels were measured using a Thermo Scientific Haake Mars 40, where the strain-sweep mode at a strain amplitude range of 0.01%–10% at a frequency of 6.28 rad/s and the frequency-sweep mode at a frequency of 1 rad/s to 60 rad/s with 1% strain. For recovery-mode, G′ and G″ were measured with a strain of 0.1% and a frequency of 6.28 rad/s for 300 s, followed by the strain was set to 300% and the frequency of 100 Hz for 60 s, and subsequently switched back to 0.1% strain and 6.28 rad/s to monitor the recovery of mechanical properties for 300 s.

#### SBF Immersion and Scanning Electron Microscopy

SBF was prepared by adding 700 ml of deionized water to a 1000 ml beaker, followed by 7.996 g of NaCl, 0.350 g of NaHCO_3_, 0.224 g of KCl, 0.228 g of K_2_HPO_4_.3H_2_O, 0.305 g of MgCl_2_.6H_2_O, 4 ml of 10 mol/L HCl, 0.278 g of CaCl_2_ and 0.071 g of NaSO_4_. After full dissolution, 6.057 g of (CH_2_OH)_3_CNH_2_ was added carefully dropwise, i.e., less than 1 g at a time. Then, 300 ml of deionized water was added and pH was adjusted to 7 with HCl. To investigate the apatite forming ability of hydrogels, fabricated SBF-PRP (-) and SBF-PRP (+) hydrogels were placed in a 12-well plate filled with 1 ml of SBF solution and incubated at 37°C for 5 days. Collected samples were washed with deionized water and lyophilized before characterizing the formed apatite by scanning electron microscopy (SEM). The microstructures and cross sections of hydrogels were captured at 3 kV by energy dispersive X-ray spectroscopy (EDS) at 8 kV.

#### Kinetice of TGF-β Release From PRP-Incorporated Hydrogels

The PRP-incorporated hydrogels [PBS-PRP (+), SBF-PRP (+)] were placed in a 12-well plate filled with 1 ml of PBS at 37°C. At pre-determined time intervals over 14 days, the PBS were taken out for measuring the amount of released TGF-β by ELISA assay and re-added the fresh PBS.

### Cell Experiments

#### Cell Culture

Rabbit bone marrow mesenchymal stem cells (rBMSCs) were used in cell culture studies. The cells were incubated in DMEM with 10% (v/v) fetal bovine serum and 1% (v/v) penicillin/streptomycin (Gibco) at 37°C in a humidified incubator in the presence of 5% CO_2_. The studies were carried out with four experimental groups, including the PBS-PRP (-), PBS-PRP (+), SBF-PRP (-) and SBF-PRP (+) groups. The four groups of hydrogels were placed in twelve-well plates separately, and then 1 ml of complete medium was added per well for pre-culture. At 80–90% confluence, the cells were trypsinized and resuspended at 1×10^5^ cells/ml. Then, 500-µL cell suspensions were seeded in each group (50000 cells/well), and the culture medium was changed every 2 days. The morphology of BMSCs was observed under an inverted microscope (LEICA DMIL) on bright field and the LAS V4.12 software was used for imaging.

#### Cell Viability Detection

Cell proliferation rate was assessed with CCK-8 (E1CK-000208; Enogene Biotech., Nanjing, China). Four groups of hydrogels were cut to fit 96-well plates, and 5,000 cells in 20 µL medium were seeded in each well. The original medium was discarded after 1, 3, 5, and 7 days of culture, respectively. Complete medium containing 10% CCK-8 solution was added to each well and placed in an incubator for 4 h. A microplate reader (BioTek ELx808) was used to detect absorbance at 450 nm.

#### Observation of Cell Morphology

The morphological features of BMSCs on PBS-PRP (-), PBS-PRP (+), SBF-PRP (-) and SBF-PRP (+) hydrogels were observed by confocal laser scanning microscopy respectively. Cells were washed with PBS twice and incubated with phalloidin and DAPI in a dark environment.

#### Live/Dead Staining

After 24 h of culture, hydrogels with BMSCs were transferred to confocal dishes and washed with PBS. Then, fluorescein diacetate (FDA)/propidium iodide (PI) mixture was applied for live/dead staining. Cells were observed and imaged with a laser scanning confocal microscope (LSM710; Zeiss, Germany) at 488 nm for living cells and 565 nm for dead cells.

#### Immunofluorescent Staining

After 7 days of osteogenic differentiation, the expression of RUNX2 in BMSCs cultured on PBS-PRP (-), PBS-PRP (+), SBF-PRP (-) and SBF-PRP (+) hydrogels was detected by IF staining. Briefly, cells were fixed with 4% paraformaldehyde at 4°C and permeabilized with 0.5% Triton X-100. After permeabilization for 10 min, cells were washed three times with PBS and blocked with 3% BSA for 30 min. Diluted anti-RUNX2 primary antibodies were added and incubated overnight at 4°C. Subsequently, cells were rinsed three times with PBST (PBS +0.1% Tween 20) and subjected to further incubation with Alexa Fluor 565-conjugated secondary antibodies for 1 h in the dark at room temperature. Fluorescence images were captured under a confocal microscope.

#### ALP Activity Assay and ALP Staining

BMSCs were seeded as described above. After incubation in osteogenic induction medium for 7 days, cells in four groups of hydrogels were harvested separately for analysis. ALP activity was assessed with the ALP/AKP assay kit (A059-2-2, Boqiao Biotech, Nanjing, China) according to the manufacturer’s instructions, normalized to total protein concentration detected with BCA Protein Assay Kit (PT0001, Leagene, Beijing, China). BCIP/NBT Alkaline Phosphatase Color Development Kit (C3206, Beyotime, Shanghai, China) was applied for ALP staining after 7 days of osteogenic induction. Cells were fixed with 4% paraformaldehyde and stained according to the manufacturer’s instructions.

#### Alizarin Red S Staining

Cells were incubated with leach liquor of four group of hydrogels, after 21 days of osteogenic differentiation, ARS staining was performed with 1% ARS (pH 4.2, Leagene, Beijing, China) after fixation with 4% paraformaldehyde. After incubation at RT for 5 min, cells were rinsed with PBS and imaged. Further quantification of calcium mineralization was detected by immersing the stained cells in 10% (w/v) cetyl pyridinium chloride for 1 h, and the absorbance was measured using a plate reader at 562 nm.

#### RNA Isolation and Quantitative Real-Time Polymerase Chain Reaction

Total RNA was extracted from BMSCs with Cell/Bacteria Kit (Tiangen Biotech Co., Ltd., Beijing, China) after 24 h of culture in complete medium and 14 days of culture in osteogenic medium respectively. qRT-PCR primers were shown in Supplementary Table S2 **.** GAPDH was used as a reference, and quantitative real-time PCR was performed on an ABI 7900 Real-Time PCR System (Thermo Fisher Scientific) with TB Grenn Premix Ex Taq II (RR820A; Takara Bio Inc., Japan) according to the manufacturer’s instructions. Relative expression levels were calculated by the 2^−ΔΔCt^ method, and each analysis included three to five replicates.

#### Western Blot

Western blot was performed as previously described (Liu et al., 2021), with primary antibodies targeting BMP2, OPN, COLI, ALP, TUBLIUN, and GAPDH (Tanon 5200).

### Animal Experiments

Twelve Male Newland rabbits with an average weight of 4 kg were obtained from the Laboratory Animal Center of Drum Tower Hospital affiliated to the Medical School of Nanjing University (China). All experimental protocols were approved by the ethics committee of Drum Tower Hospital affiliated to the Medical School of Nanjing University, and performed according to the Institutional Animal Care and Use Committee (IACUC) guidelines.

#### Femur Condyle Defect Model

New Zealand rabbits were anesthetized with propofol and lidocaine, followed by the establishment of a 5-mm defect in the lateral femur. Then, SBF-PRP (+) and SBF-PRP (-) hydrogels were implanted in the experimental and control groups, respectively. No operation was performed in the blank control group. Femur condyles were harvested at eight postoperative weeks for further bone tissue regeneration evaluation.

#### Micro-CT Analysis and 3D Reconstruction

The harvested femur condyles were scanned on a vivaCT 80 system (V6.5-3 Scanco Medical, Bruettisellen, Switzerland). The operating voltage and current were 45 KeV and 145μA, respectively. Bone mineral densities and relevant bone trabecula parameters were obtained according to micro-CT data. Three-dimensional models of the harvested femur condyles were reconstructed with MIMICS 19.0 (Materialise, Leuven, Belgium).

### Statistical Analysis

All experiments were performed with three replicates unless otherwise stated. Data are mean ± SD. Statistical analysis was performed with the Origin software (8.5 version). Asterisks in statistical analysis indicate statistically significant differences between the control and experimental groups (∗*p* < 0.05; ∗∗*p* < 0.01; ∗∗∗*p* < 0.005; ∗∗∗∗*p* < 0.001).

## Resluts and Discussion

### Design of the PRP-Hydrogel

**FIGURE 1:**
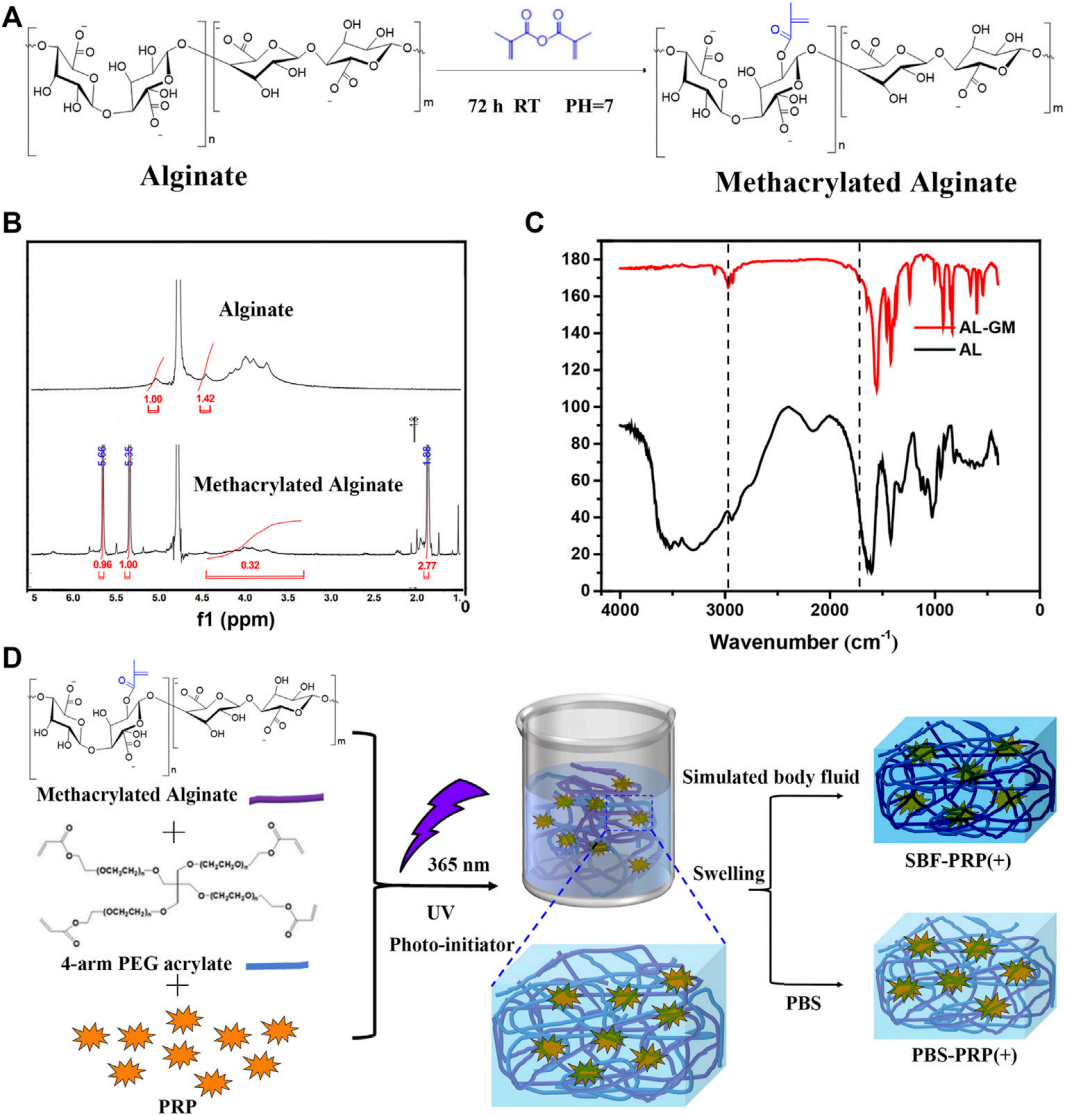
Preparation of IPN M-ALG/PEG hydrogels. **(A)** Reaction schemes, **(B)** 1H-NMR spectra and **(C)** FTIR spectra of M-ALG. **(D)** Schematic of preparation process of IPN M-ALG/PEG hydrogels alone or combine with PRP.

Methacrylated alginate (M-ALG) was synthesized from the reaction between the hydroxyl group of alginate and the epoxy group of methacrylic anhydride under alkaline conditions, the mechanisms of which include epoxide ring-opening, carbodiimide chemistry and transesterification (Araiza-Verduzco et al., 2020). The reaction is schematically shown in Figure 1A, and the structures of neat ALG and M-ALG were confirmed by 1 HNMR spectroscopy in D_2_O (Figure 1B), depicting characteristic peaks between 3.50 and 5.20 ppm from both neat ALG and M-ALG due to their saccharide units. As expected, the spectrum of M-ALG displayed distinctive peaks corresponding to the vinyl (5.35 and 5.66 ppm) and methyl (1.80 ppm) hydrogens of methacrylate grafted groups, consistent with previous reports (Wang et al., 2015). Of note, these peaks could slightly shift due to different chemical environments (Burdick et al., 2005). Furthermore, the chemical structures of neat ALG and M-ALG were characterized by FTIR. As shown in Figure 1C, M-ALG′ spectrum exhibited two additional bands compared with that of ALG (arrows): occurrence of–CH stretching bands (2980–2850 cm^−1^) and appearance of a shoulder (1721 cm^−1^), which were attributed to stretching vibrations of the aliphatic chains’ –CH groups and the esters’ C=O group, respectively. Both groups were due to the grafting of methacrylate units (Mugnaini et al., 2021) (chemical structures in Figure 1A). These results confirmed that methacrylate was successfully grafted to neat ALG.

**FIGURE 2:**
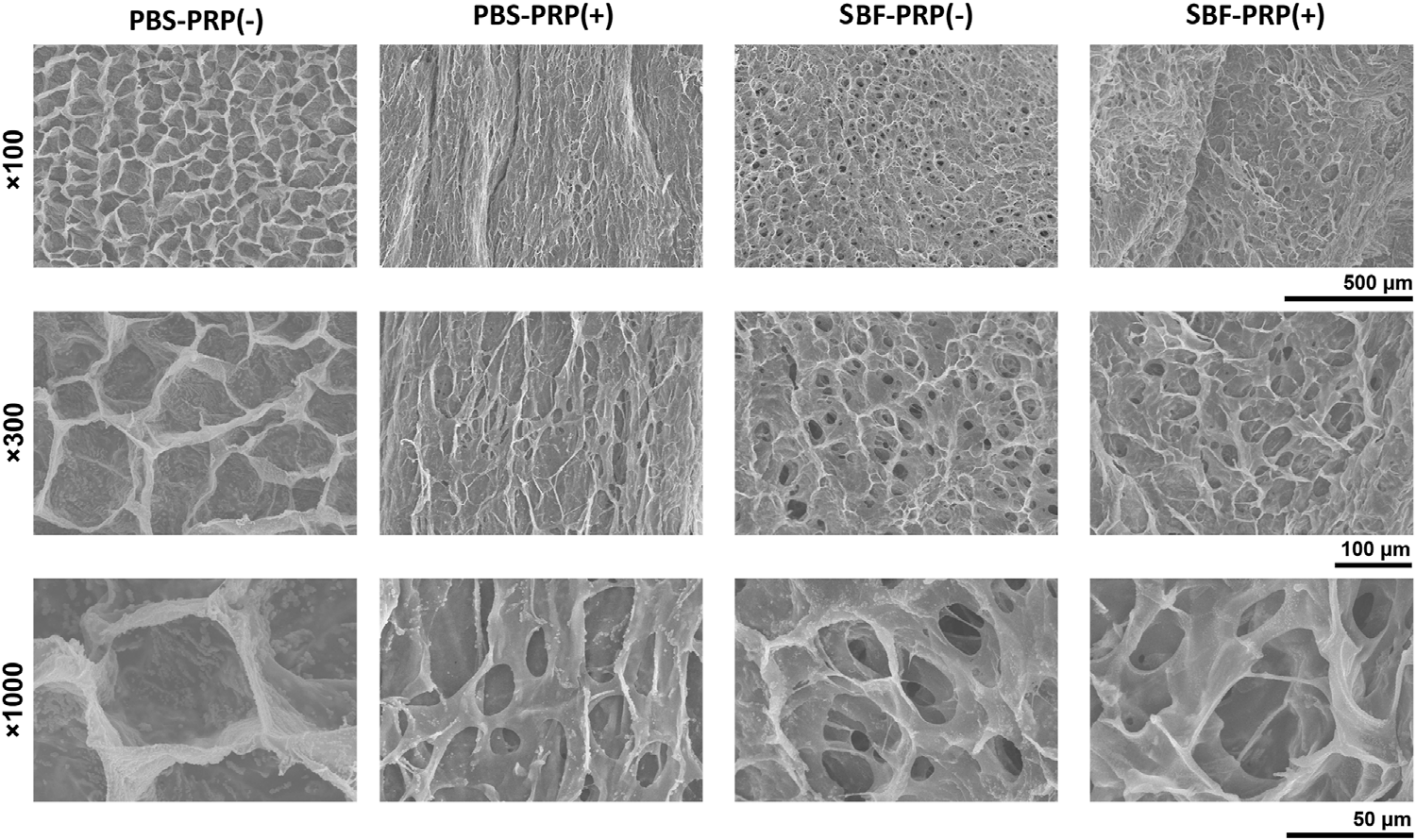
Surface topography and their local magnification of IPN M-ALG/PEG hydrogels observed by SEM.

PRP-incorporated interpenetrating polymeric network (IPN) hydrogels were synthesized through free radical polymerization reaction under UV-light irradiation in the presence of PRP (Figure 1D). Covalent crosslinks between M-ALG (methacrylate groups) and 4-Armed PEG-ACLT (acrylate groups) were formed after this reaction, where a biocompatible photoinitiator (I2959) was used to induce fluid-solid phase transformations. Tetra-functionalized PEG and M-ALG crosslinkers were used due to their high crosslinking efficiency, and the addition of PRP improved biological activities (Andersen et al., 2021; Xu et al., 2021). The as-prepared hydrogels were further crosslinked by exposure to SBF that contained Ca^2+^ to interact with ALG, forming mineralized PRP-incorporated IPN hydrogels. Photographs of as-prepared hydrogels, including PBS-PRP (-), PBS-PRP (+), SBF-PRP (-) and SBF-PRP (+) were shown in Supplementary Figure S1, depicting the hydrogel turning pale yellow after the incorporation of PRP. As expected, the volume of the hydrogel alone or combined with PRP biomineralized by SBF was smaller than that of the corresponding hydrogel treated with PBS, indicating that biological mineralization hindered the swelling of hydrogels. In addition, lyophilized PRP-incorporated IPN hydrogels were morphologically characterized by SEM. As illustrated in Figure 2, all IPN hydrogels had a porous structure with a pore size of about 20–50 μm. Different from the uniform pore size in the PBS-PRP (-) group, pore size in the PBS-PRP (+) group was slightly discrepant, and its surface appears clearly cross-linked fibers, indicating the formation of a platelet activation-dependent fibrin network which is consistent with the results of previous studies (Qian et al., 2022). In contrast to the PBS-PRP (-) group, hydrogel pores in the mineralized group are smaller while the pore wall thickness is significantly increased, which is related to the presence of more crosslinks within the scaffold of the mineralized group. Furthermore, EDS mapping based on SEM images was also present to verify the biological mineralization between Ca^2+^ in SBF and ALG. As shown in Supplementary Figure S2 and Supplementary Table S1, all IPN hydrogels contained O, Na, Cl and K. Meanwhile, Ca was only presence in SBF-PRP (-) and SBF-PRP (+), which was attributed to successful biological mineralization. In particular, we expected the porous microstructures are suitable for containing of cells, thereby accommodating a large number of cells and facilitating multiple cellular functions, including cell attachment, proliferation and differentiation (Li L. et al., 2020; Huang et al., 2021).

### Physicochemical Properties of the PRP-Hydrogel

**FIGURE 3:**
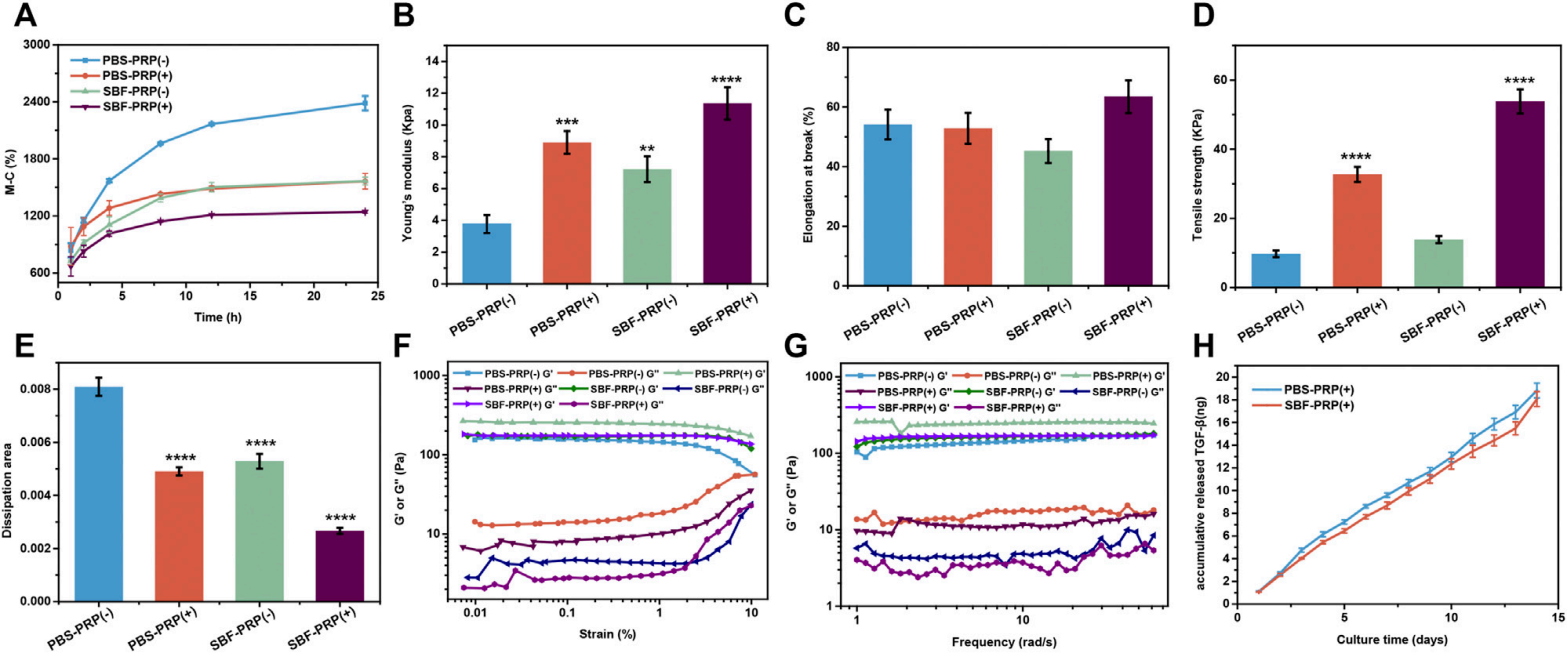
Properties of IPN M-ALG/PEG hydrogels. **(A)** Swelling rate at different time from 1 to 24 h. **(B)** Young’s modulus, **(C)** Elongation in break, **(D)** Tensile strength and **(E)** dissipation area of IPN M-ALG/PEG hydrogels. **(F)** G′ and G″ of the IPN M-ALG/PEG hydrogels measured in a strain sweep experiment (from 0.1 to 10% strain, 6.28 rad s^−1^) at room temperature. **(G)** G′ and G″ of the IPN M-ALG/PEG hydrogels measured in a frequency sweep experiment (from 0.1 to 60 rad s^−1^, 1% strain) at room temperature. **(H)** TGF-β release in PBS buffer from 0 to 14 days of PRP-IPN hydrogels.

It is known that bioactive materials used as bone repair scaffolds should possess several features, including high surface area/volume ratio, proper mechanical properties, excellent biocompatibility and enhanced osteoconductivity (Dong et al., 2019; Wang et al., 2020). By achieving these, scaffolds can provide cells with an appropriate three-dimensional (3D) environment, enabling cells to exert normal functions such as proliferation and migration, and subsequently support the newly formed bone (Sun et al., 2016; Zhu et al., 2021). Hence, the physicochemical properties of the as-prepared IPN hydrogels were assessed. Swelling rate, one of the most important properties that can reflect the crosslinking degree, affinity toward H_2_O molecules, ionization degree of functional groups and properties of swelling medium such as ionic strength and temperature (Kim et al., 2013) were firstly measured. As shown in Figure 3A, swelling rate decreased after the biological mineralization process, suggesting ionic crosslinking between Ca^2+^ and ALG occurred. Furthermore, we also studied the compressive mechanical properties of as-prepared IPN M-ALG/PEG hydrogels by standard mechanical tests. The obtained stress-strain curves based on the compression-crack test were shown in Supplementary Figure S3A, which indicated that SBF-PRP (+) demonstrated the enhanced Young’ modulus (11.36 ± 1.02 Kpa) compared with the other hydrogels (Figure 3B). Strikingly, the elongations at break (Figure 3C) and tensile strength (Figure 3D) of hydrogels increased after the PRP incorporation and the biological mineralization, which substantially improved the load bearing ability of the hydrogel. Furthermore, the energy dissipation levels of hydrogels were investigated through compression-relaxation cycles under 50% strain. As shown in Supplementary Figure S3B, all hydrogels demonstrated slight energy dissipation, with no significant difference among them, which could be attributed to the rupture of physical cross-linkers. As expect, the calculated dissipation of hydrogels (Figrue 3E) was significantly decreased after the incorporation of PRP and the biological mineralization process. The fast recovery ability of hydrogels was then tested by applying continuous compression-relaxation cycles for 10 cycles (Supplementary Figure S3C). The results revealed that the tensile strength of each cycle was slightly decreased but remained at more than 85% after 10 continuous cycles, suggesting the remarkably fast recovery ability of IPN hydrogels. In next, the rheological properties of the as-prepared hydrogels were investigated. Strain-dependent oscillatory shear rheology (Figure 3F) exhibited a strain at yield strength of 10%, which was the cross point of G′ and G″, representing the transition of the hydrogel from solid to liquid state. Besides, the frequency-sweep test (Figure 3G) demonstrated that the storage modulus (G′) was larger than the loss modulus (G″), suggesting that the fabricated hydrogels exhibited a solid-like behavior. Furthermore, step-strain measurements (Supplementary Figure S4) demonstrated that all hydrogels were destroyed partly and subsequently recovered completely and rapidly from a strain of 300%–0.1% for four cycles of breaking and reforming, revealing the dominant elastic nature of these hydrogels. Furthermore, the accumulative release of TGF-β from PRP-incorporated IPN hydrogels was investigated in phosphate buffer saline (PBS) over an incubation period of 14 days, which was quantified by enzyme-linked immuno sorbent assay (ELSA). The obtained release curves are shown in Figure 3H, sustained release of TGF-β over the investigated period could be observed for both PBS-PRP (+) and SBF-PRP (+). As expect, SBF-PRP (+) demonstrated relatively slower release due to the biological mineralization layer partly prevented the release of TGF-β. These results indicated that the as-prepared PRP-incorporated IPN hydrogels possess substantially improved mechanical strength and sustained release of growth factor, constituting potential repair scaffolds for bone tissue regeneration.

### Proliferation and Attachment of BMSCs on the PRP-Hydrogel

**FIGURE 4:**
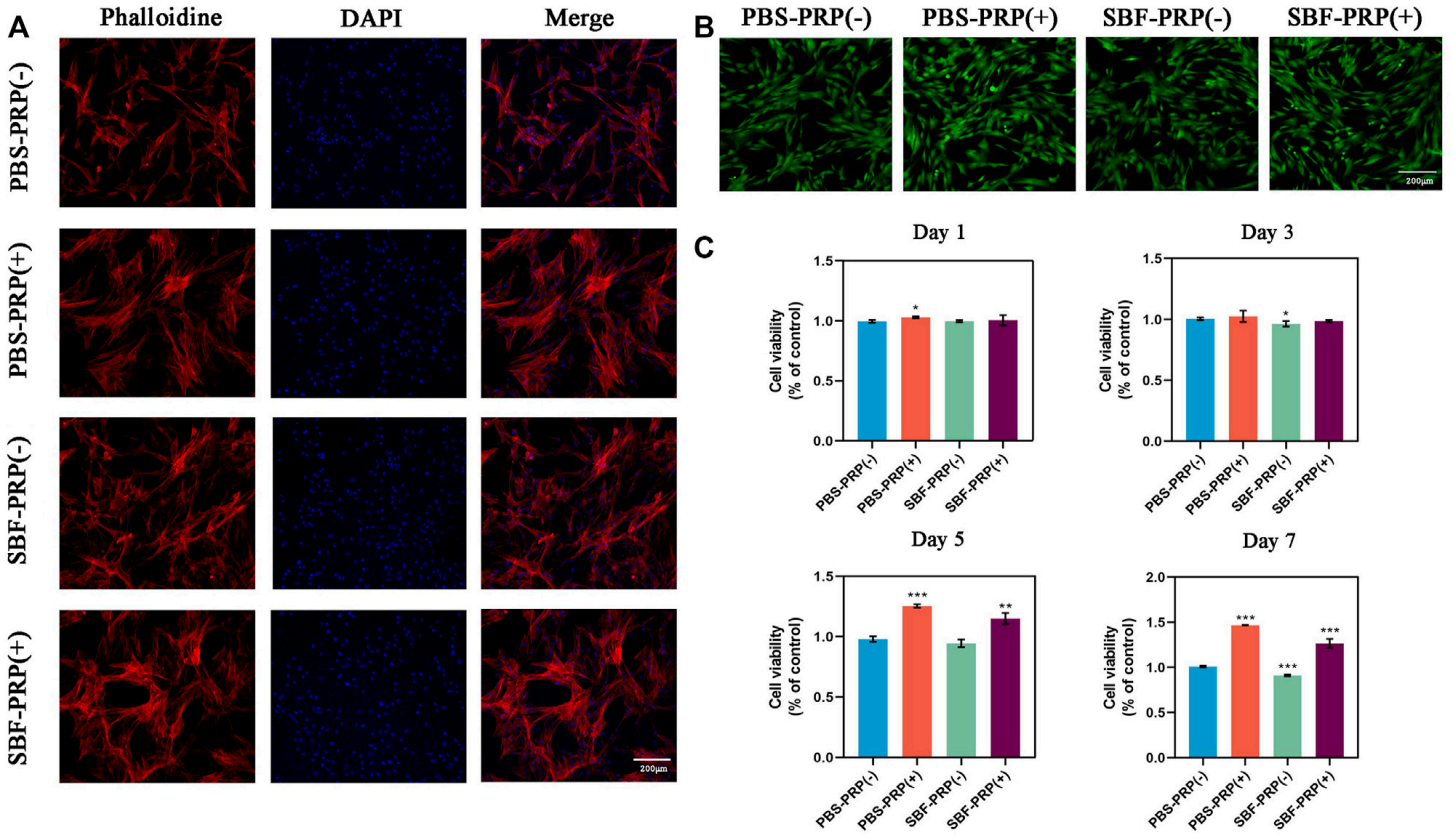
Biocompatibility of as-prepared hydrogels. **(A)** Cytoskeleton of BMSCs cultivated on PBS-PRP (-), PBS-PRP (+), SBF-PRP (-) and SBF-PRP (+) hydrogels respectively. **(B)** Live/Dead staining of BMSCs after inoculated on four groups of hydrogels for 24 h. **(C)** Cell viability of BMSCs after cultivated on four groups of hydrogels for 1, 3, 5 and 7 days respectively. Asterisk indicates statistically significant differences between control and experimental group (∗*p* < 0.05; ∗∗*p* < 0.01; ∗∗∗*p* < 0.005).

The physical characterization of the scaffold was followed by cytocompatibility assessment *in vitro*. It is known that the integration of scaffolds with the natural bone by forming the apatite layer on its surface *in vivo* can be simulated *in vitro* by scaffold incubation in SBF (Lewandowska-Łańcucka et al., 2019). Considering the possible *in vivo* application of this scaffold in BTE, *in vitro* biomineralization of the PRP-hydrogel scaffold was performed using SBF. Subsequently, the cytocompatibility of scaffolds was assessed by co-incubating with cells and measuring cell viability, cell proliferation and cell adhesion assays. Cell morphology on different groups was depicted in Figure 4A. As demonstrated, cells can well spread, survive, and maintain their spindle-shaped morphology on the hydrogels, suggesting the excellent cytocompatibility of both non-mineralized and mineralized scaffolds (Figure 4B). It is worth to mention that higher cell attachment was found after 24 h of cell incubation on both non-mineralized and mineralized scaffolds in the presence of PRP compared with the control group. Also, cell morphology observation on all four groups after 48 h incubation was obtained by optical microscopy and confocal microscopy (Supplementary Figure S6). These results demonstrated that the incorporation of PRP facilities cell adhesion. In order to further evaluate the effect of PRP on cell adhesion, RT-PCR was performed to detect the expression of integrin β1, which is a classic transmembrane receptor that mediate the attachment between a cell and its surroundings. RT-PCR primer of integrin β1 was shown in revised Supplementary Table S2. BMSCs were cultivated on PBS-PRP (-), PBS-PRP (+), SBF-PRP (-) and SBF-PRP (+) hydrogels for 24 h and then collected respectively. Then the relative expression of integrin β1 was detected by RT-PCR. As shown in the revised Supplementary Figure S5, cells seeded on the PRP-containing hydrogels exhibit higher expression of integrin β1, with or without the presence of SBF. This is consistent with our previous results obtained by cytoskeleton staining in Figure 4A. Cell proliferation on the scaffolds was further quantified by the CCK-8 assay after 1, 3, 5, and 7 days of cell culture, respectively. As shown in Figure 4C, nearly no significant difference was observed at 1 and 3 days and the numerical results were shown in Supplementary Table S3 **.** Surprisingly, PRP-incorporated IPN hydrogel scaffolds had a higher cell proliferation rate than the neat hydrogel scaffolds after 5 and 7 days of culture (*p* < 0.05), which indicated that the incorporation of PRP into the alginate/ethylene-glycol hydrogel greatly promotes BMSC proliferation. In general, multiple studies have consistently confirmed that PRP increase cell proliferation *in vitro* (Gentile and Garcovich 2020), and this effect is mainly attributed to the release of growth factors from platelets (Martino et al., 2011). In this study, benefiting from the novel double-network structure of the hydrogel scaffold, biomolecules from PRP could more readily penetrate the scaffold and be continuously released without being affected by the mineralization of scaffolds.

### Effects of the PRP-Hydrogel on Osteogenic Differentiation of BMSCs

**FIGURE 5:**
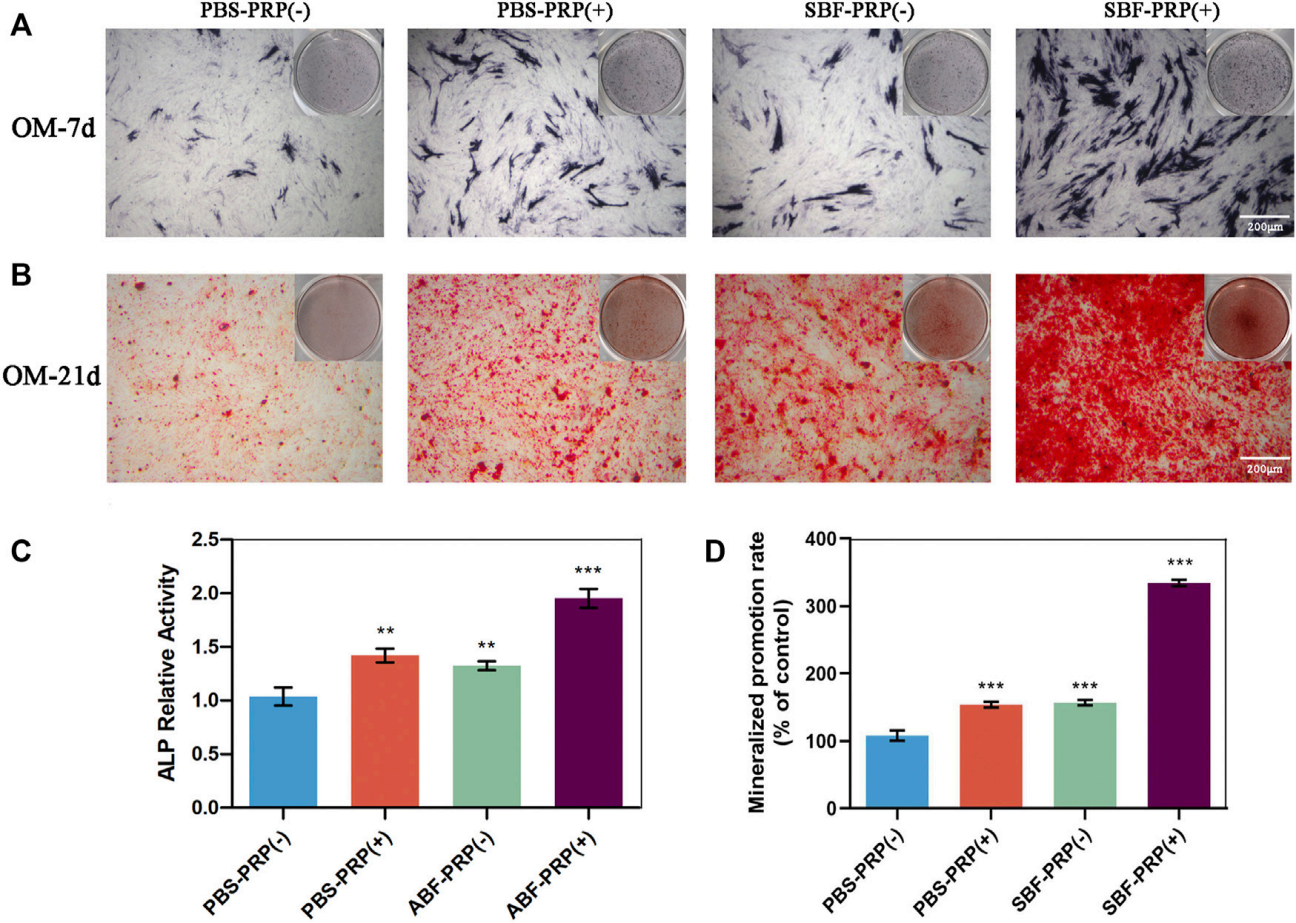
ALP activity and calcium nodule production of BMSCs. **(A)** ALP staining of BMSCs after osteogenic differentiation for 7 days. **(B)** Alizarin red staining of BMSCs after osteogenic differentiation for 21 days. **(C)** ALP activity level of BMSCs after osteogenic differentiation for 7 days. **(D)** Quantification of Alizarin red staining in **(B)**. (∗*p* < 0.05; ∗∗*p* < 0.01; ∗∗∗*p* < 0.005).

**FIGURE 6:**
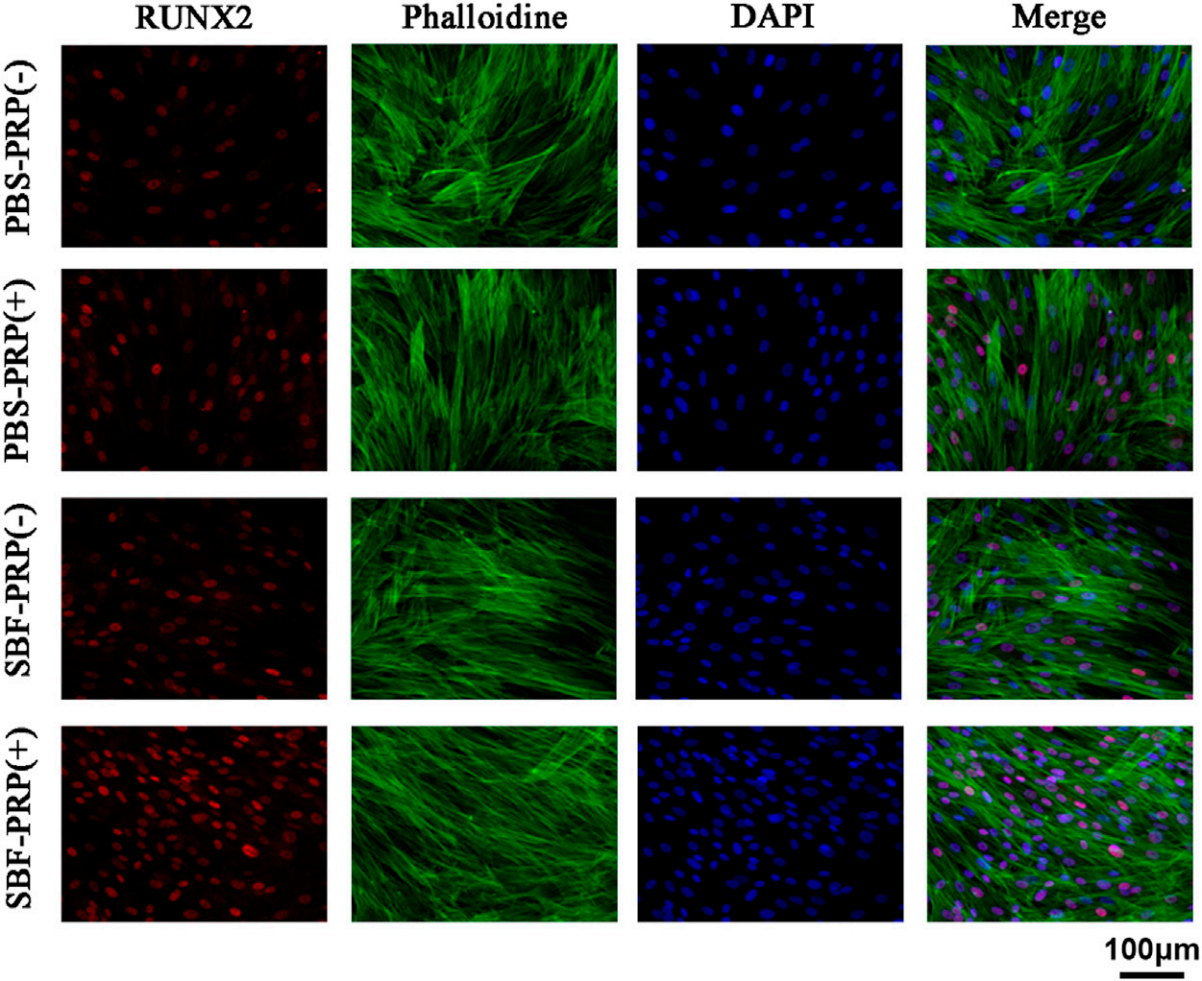
RUNX2 expression of BMSCs cultivated on PBS-PRP (-), PBS-PRP (+), SBF-PRP (-) and SBF-PRP (+) hydrogels. RUNX2, F-actin and nuclear were visualized by RUNX2-specific antibodies (red), rhodamine phalloidin (green) and DAPI (blue) respectively.

**FIGURE 7:**
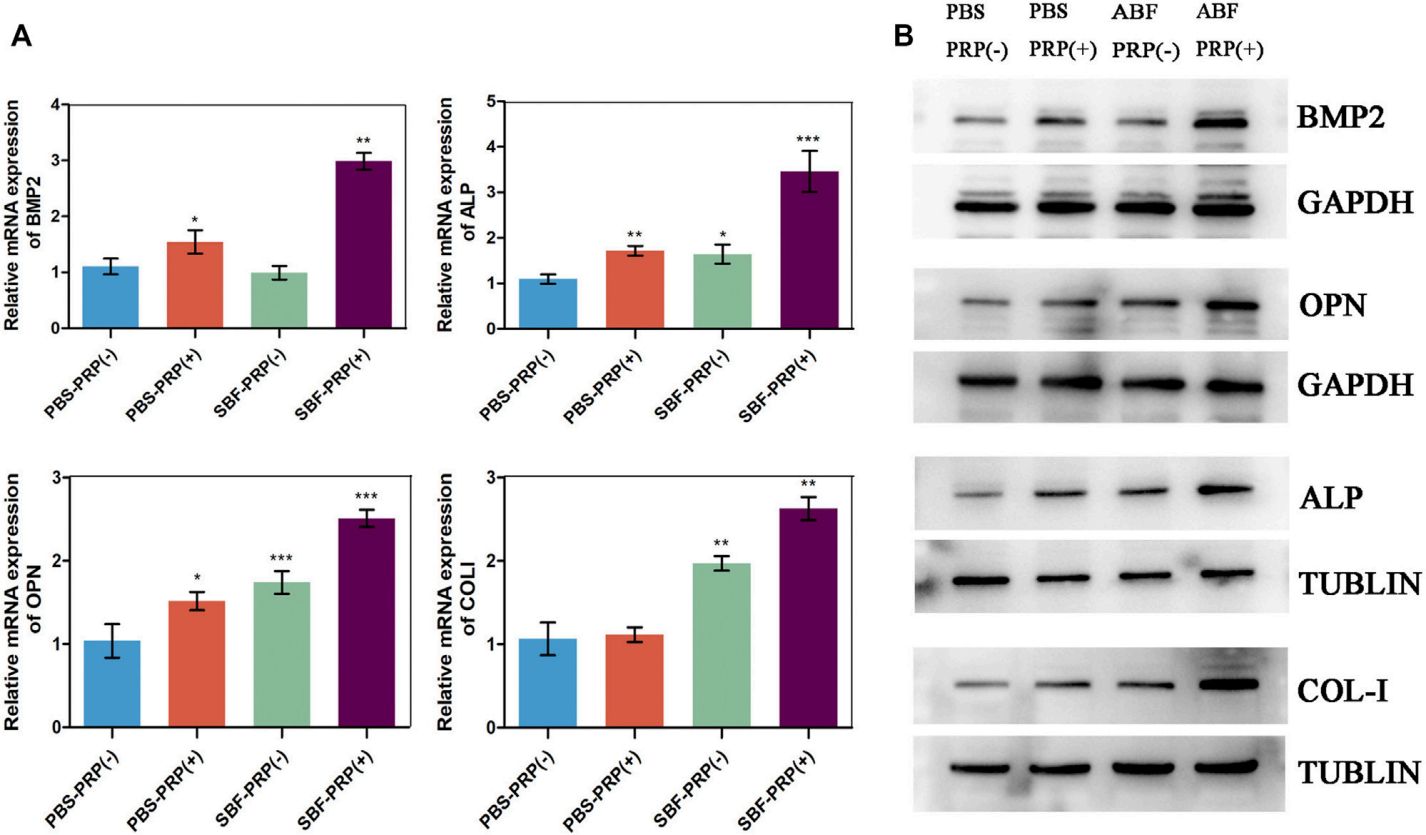
Osteogenic gene and protein expression of BMSCs**. (A)** RT-PCR analysis of BMP2, OPN, ALP and COLI in BMSCs after 14 days cultivation on PBS-PRP (-), PBS-PRP (+), SBF-PRP (-) and SBF-PRP (+) hydrogels. **(B)** Western blot analysis of BMP2, OPN, ALP and COLI in BMSCs cultivated on PBS-PRP (-), PBS-PRP (+), SBF-PRP (-) and SBF-PRP (+) hydrogels. *p < 0.05 **p < 0.01 ***p < 0.001 (Student’s t-test). Data are presented as mean ± SD (n ≥ 3).

ALP activity and ARS staining intensity were detected after seeding of BMSCs. Higher ALP activity (Figures 5A,C) and ARS intensity (Figures 5B,D) were quantitatively and statistically found on the BMSCs cultured on mineralized PRP-containing substrate compared with others, indicating an increased osteogenic differentiation capacity. We then cultured BMSCs on scaffolds and analyzed RUNX2 expression by immunofluorescence (IF) (Figure 6). Increased amount of RUNX2-positive cells were observed in the SBF-PRP (+) group compared with the other groups. Furthermore, we analyzed the expression levels of common osteogenesis markers (Tang et al., 2017), including BMP2, OPN, ALP and COL-I, and found that osteogenesis-related genes were significantly upregulated at both mRNA and protein levels in BMSCs cultured on the mineralized PRP-hydrogel scaffold (Figure 7). The raw data of western blot analysis was shown in Supplementary Figure S7 and semi quantitative analysis of western blot was shown in Supplementary Figure S8. These data demonstrated that PRP and mineralization of the scaffold synergically promote osteogenic differentiation in BMSCs. However, the osteogenic effects of PRP are complex. Some studies have suggested that PRP may facilitate bone formation in combination with MSCs from different species; others have shown that PRP decreases the osteogenic differentiation of MSCs (Gruber et al., 2004; Felka et al., 2010). This is probably because PRP highly varies from donor to donor, and each PRP preparation may differ in the concentrations of proteins and growth factors (Lim et al., 2013). Recent research reported that PRP, in a certain concentration range, promotes cell proliferation at the early stage of differentiation, but causes no impairment of osteogenic differentiation in BMSCs (Xu et al., 2015; Xie et al., 2020). This is partly consistent with our findings that PRP had a positive effect on the proliferation of BMSCs on PRP-hydrogel scaffolds whether mineralized or not, and stimulated the osteogenic differentiation of BMSCs, particularly those cultured on the mineralized PRP-hydrogel. It is known that differentiation and proliferation represent dichotomous aspects of cellular function, and proliferation is frequently associated with differentiation (Schröder et al., 2009; Zheng et al., 2021a; Zheng et al., 2021b). A possible explanation for our findings is the sustained release of PRP from the hydrogel, which has a beneficial effect on cell proliferation and the early stage of osteogenesis. Furthermore, the biomineralization of scaffolds has been shown to considerably promote osteogenic differentiation of stem cells (Kim et al., 2021). Because the PRP-hydrogel mineralizes in a manner similar to biological mineralization, this scaffold modification significantly enhanced osteogenic differentiation of BMSCs. Summary results of the abovementioned studies lead to the conclusion that such synergistic effects of physical structures, the double-network structure/biomineralization of scaffolds, and molecular supplements (PRP) could significantly promote osteogenesis and subsequent bone regeneration.

### *In vivo* Osteogenic Effects of the PRP-Hydrogel

**FIGURE 8:**
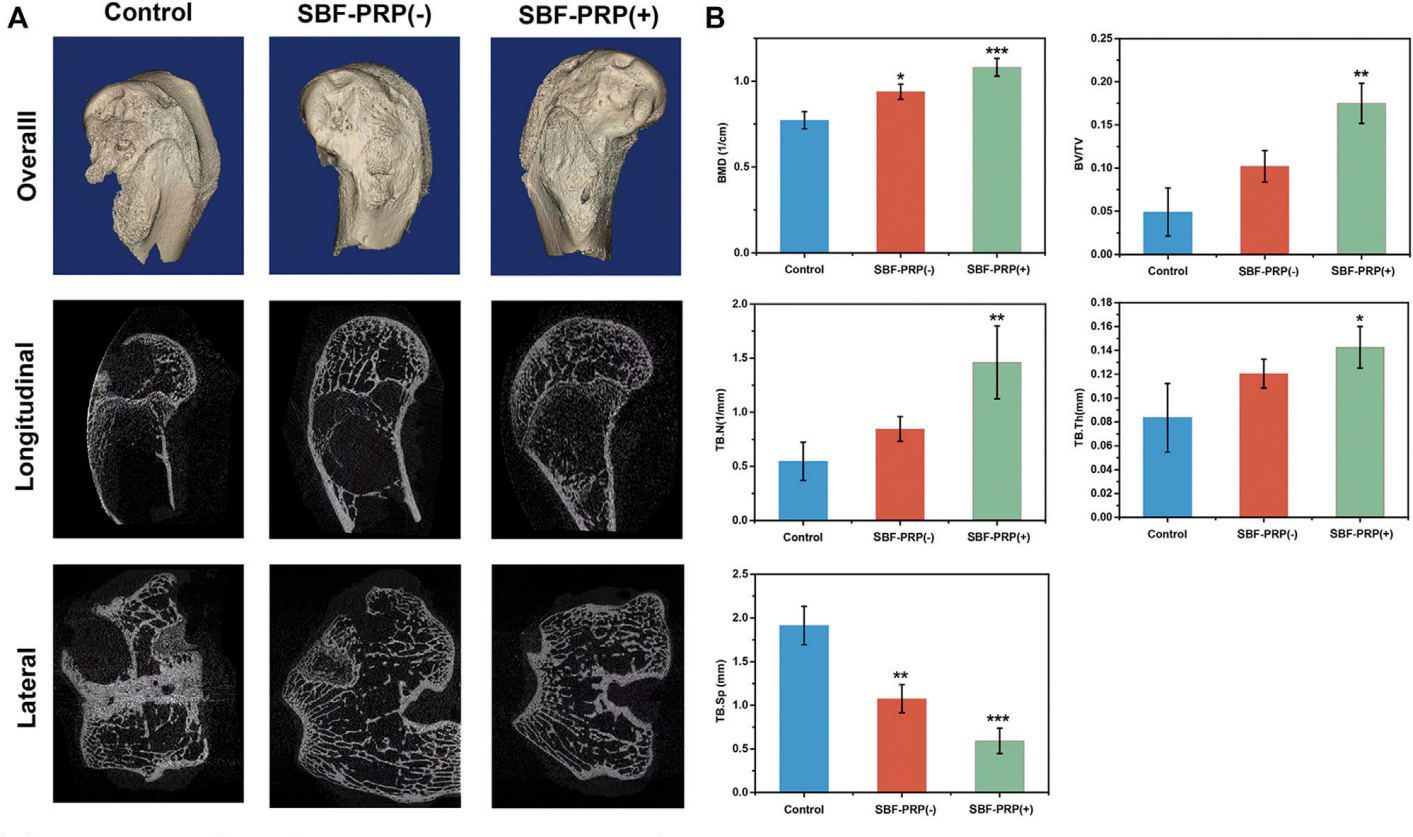
*In Vivo* osteogenic assessment of IPN M-ALG/PEG hydrogels. **(A)** Reconstructed 3D micro-CT and sectional images of femoral condyle at 8 weeks after treated with IPN M-ALG/PEG hydrogels. **(B)** Quantitatively evaluation of regenerated area by the analyzing parameter of micro-CT: BMD, BV/TV, TB.N, TB.Sp and TB.Th.

*In vitro* investigations have shown that SBF-PRP (+) hydrogel exhibited excellent biocompatibility and mechanical strength, and enhanced osteogenic activities. This prompted us to assess the *in vivo* bone regeneration performance of the fabricated hydrogels. Hence, we further evaluated the bone repair ability hydrogel with or without SBF-PRP in a rabbit model of femoral 5-mm defect (Zhang et al., 2021). SBF-PRP (-) and SBF-PRP (+) hydrogels were directly implanted into the femur defects of rabbits. For comparison, no operation was performed after creating the bone defects. At 2 months after the implantation of hydrogels, femurs were harvested and firstly observed by micro-CT. The reconstructed three-dimensional (3D) and sectional images of femoral condyles were shown in Figure 8A, indicating that there was still a large defect in the control group, while treatment with the SBF-PRP (+) hydrogel exhibited more newly formed cancellous bone compared with the SBF-PRP (-) group. For quantitative analysis, BMD, bone volume per total volume (BV/TV) and the trabecular parameters of the cancellous bone, including trabecular number (Tb.N), trabecular thickness (Tb.Th) and trabecular Spacing (TB.Sp) were assessed and the results were shown in Figure 8B. BMD in the control group was 0.77 ± 0.05 cm^−1^, while the SBF-PRP (+) group had a value of 1.08 ± 0.05 cm^−1^, which was higher than that of the SBF-PRP (-) group. A similar trend was observed for BV/TV. As for trabecular parameters, TB.N and TB. Th were significantly increased after treatment with the SBF-PRP (+) hydrogel compared with the SBF-PRP (-) and control groups, while TB. Sp was significantly decreased. These results confirmed that the SBF-PRP (+) hydrogel accelerates bone regeneration *in vivo*. Similar trends were observed in Masson staining. Taken together, these results suggested that the PRP-incorporated IPN hydrogels can rapidly and effectively promote bone regeneration.

## Summary

In summary, we reported a novel PRP-incorporated IPN hydrogel, which fabricated by combining M-ALG and 4A-PEGAcr through photo-crosslinking upon exposure to long-wave UV light and ionic crosslinked through exposure to native calcium ions in SBF. The as-prepared hydrogels exhibited expected improvement in terms of morphological and mechanical properties after PRP incorporation and biological mineralization. *In vitro* studies demonstrated that the incorporation of PRP endowed the hydrogels with excellent biocompatibility and osteogenic bioactivity, as evident by enhanced ALP activity, mineralized nodule formation, and osteogenic gene and protein expression. *In vivo* studies confirmed that the PRP-incorporated IPN hydrogels showed great ability in inducing bone regeneration. Taken together, it is anticipated that the PRP-incorporated biomineralized IPN hydrogels might be promising scaffolds for bone tissue regeneration.

## Data Availability

The original contributions presented in the study are included in the article/Supplementary Material, further inquiries can be directed to the corresponding author.
