# Supplementary material for: A Biomimetic Platelet-Rich Plasma-Based Interpenetrating Network Printable Hydrogel for Bone Regeneration
Source: Front Bioeng Biotechnol. 2022 Apr 12;10:887454. doi: 10.3389/fbioe.2022.887454 (PMC9041706; doi:10.3389/fbioe.2022.887454)
Supplement: Supplementary file 1 [file DataSheet1.docx]

**Supporting Information**

**A Biomimetic Platelet-rich Plasma-based Interpenetrating Network Hydrogel for Bone Regeneration**

***Shijia Tang^1†^, Lin Wang^1†^, Yunyang Zhang^2^, Feimin Zhang^1*^***

^1^ Jiangsu Province Key Laboratory of Oral Diseases, Department of Prosthodontics, The Affiliated Stomatological Hospital of Nanjing Medical University, Nanjing 210009, China

^2^ Center of Modem Analysis, Nanjing University, Nanjing 210008, China

^†^ Shijia Tang and Lin Wang contributed equally to this work, regarding as the first author.


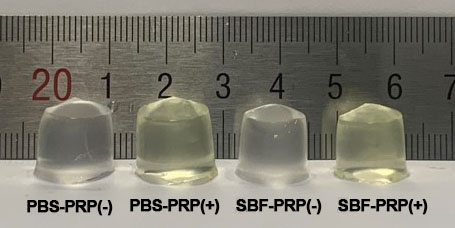


**Figure S1.** Photographs of as-prepared IPN M-ALG/PEG hydrogels.

**
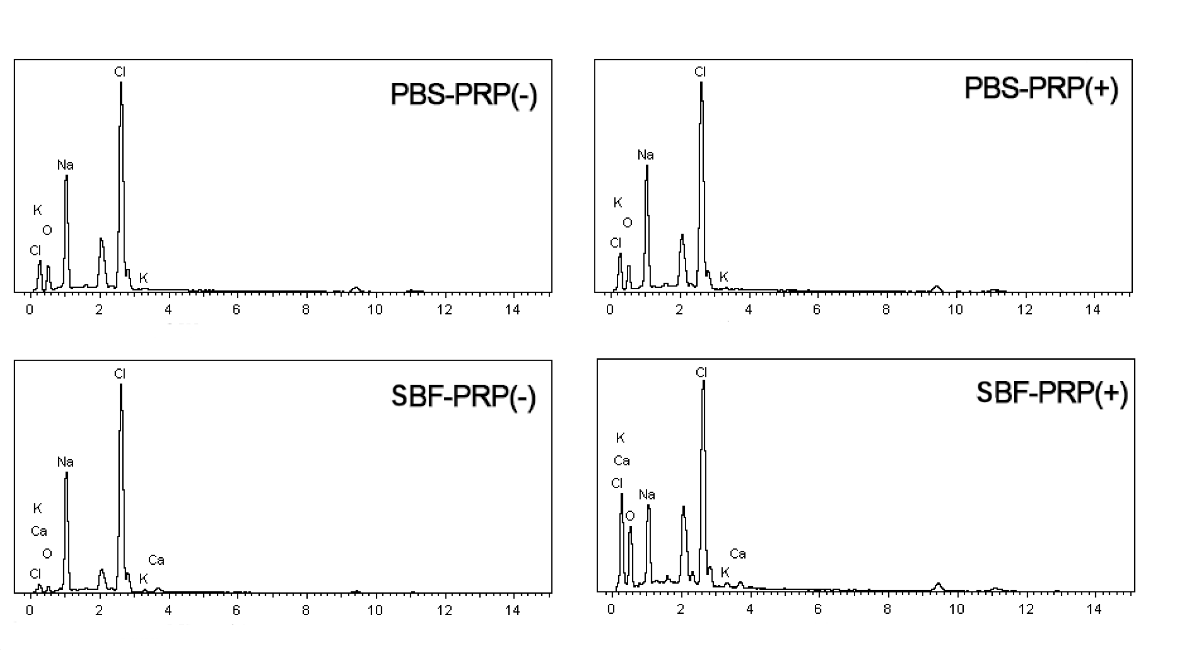
**

**Figure S2.** EDX spectra of the as-prepared IPN M-ALG/PEG hydrogels

**
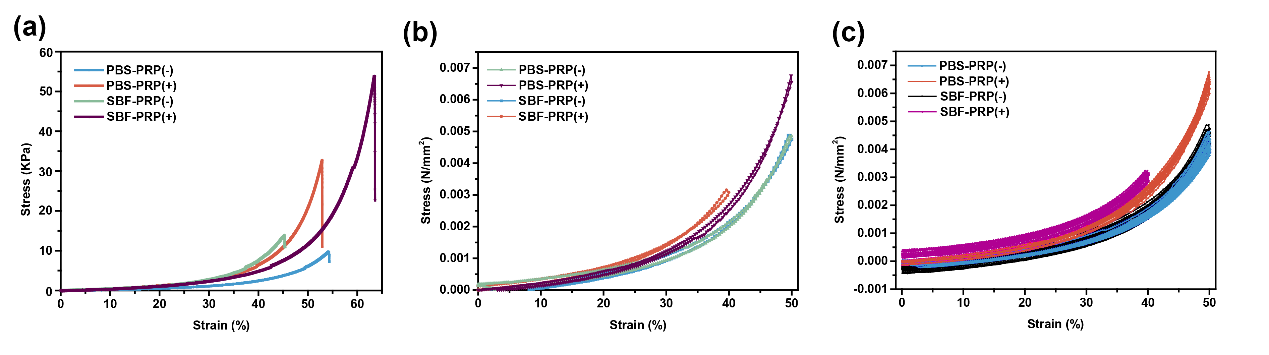
**

**Figure S3.** (a) uniaxial stress-strain curves under compression until cracking. (b) uniaxial compression-relaxation curves and (c) uniaxial compression-relaxation cycles of IPN M-ALG/PEG hydrogels.

**
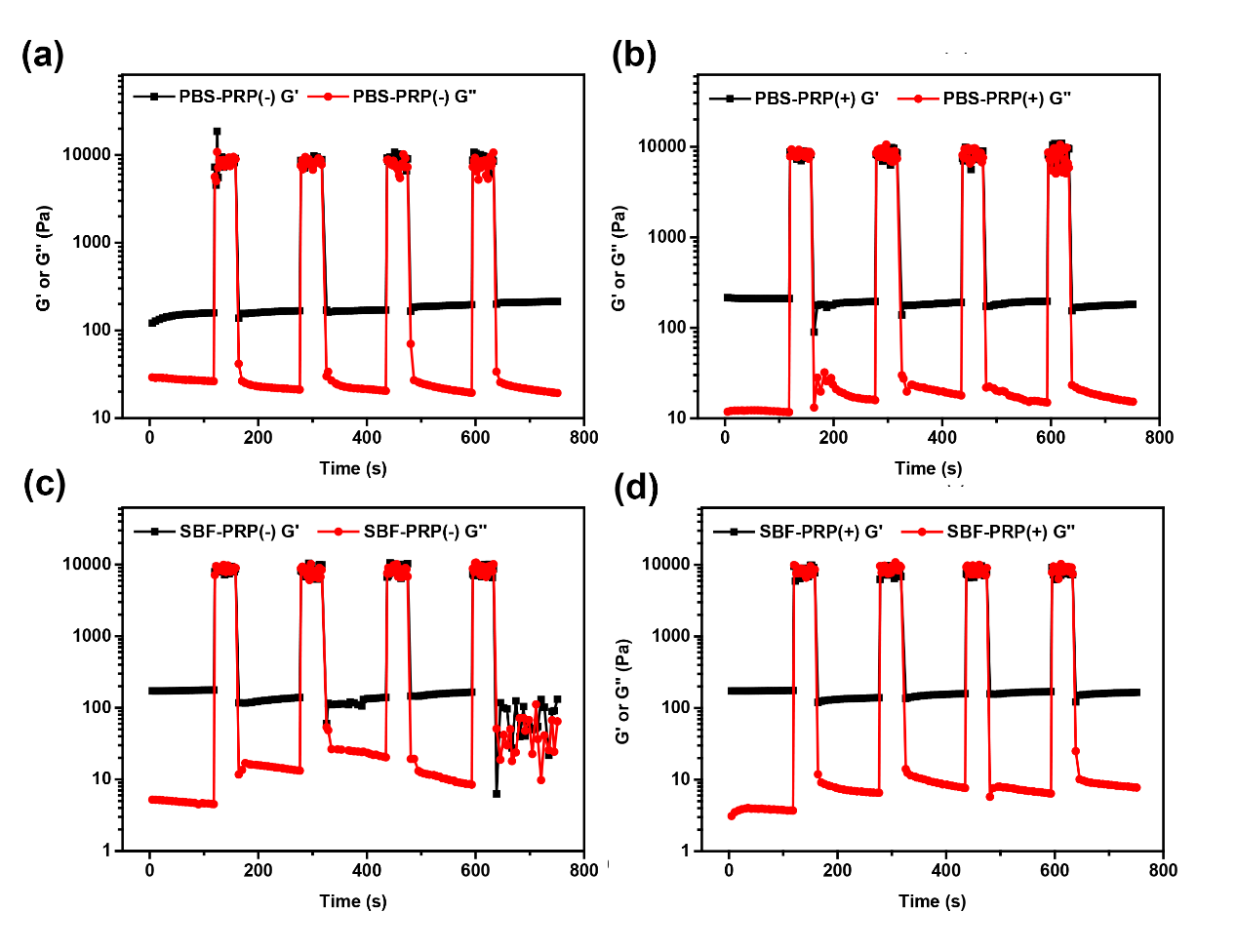
**

**Figure S4.** IPN M-ALG/PEG hydrogels in a destroy-recovery experiment at room temperature. The frequency and strain were set to the frequency of 100 Hz and amplitude of 300% to destroy the coordination interactions and switched back to a frequency of 6.28 rad s^-1^ and amplitude of 0.1% to monitor recovery of the mechanical properties.


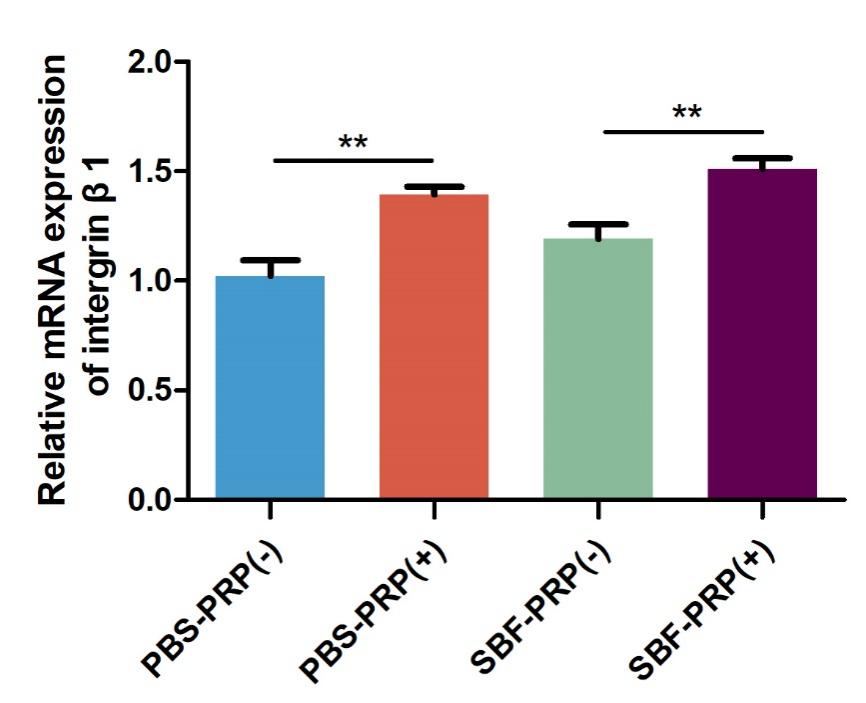


**Figure S5.** RT-PCR analysis of integrin β1 in BMSCs after 24 hours cultivation on PBS-PRP(-), PBS-PRP(+), SBF-PRP(-) and SBF-PRP(+) hydrogels.


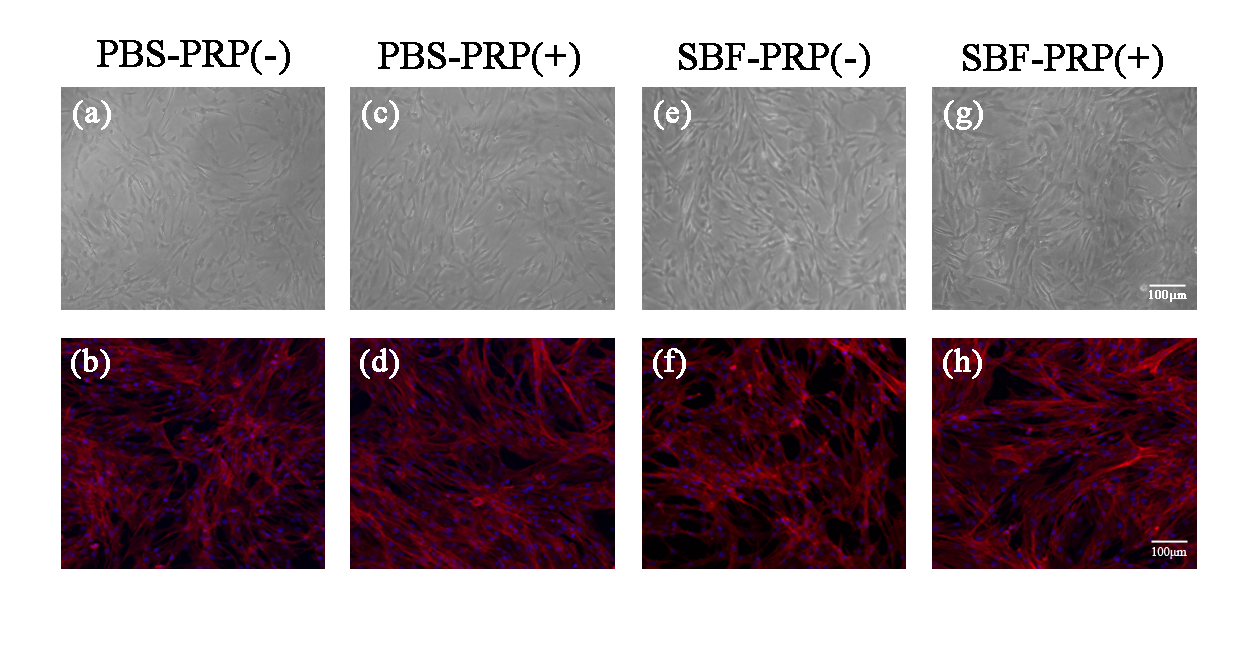


**Figure S6**. **Morphology of cells cultivated on four group of hydrogels.** Representative optical microscope and confocal microscope imagines of BMSCs cultivated on PBS-PRP **(a, b)**, PBS-PRP(+) **(c, d)**, SBF-PRP(-) **(e, f)** and SBF-PRP(+) **hydrogels (g, h)**. F-actin and nuclear were visualized by rhodamine phalloidin (red) and DAPI (blue) respectively.


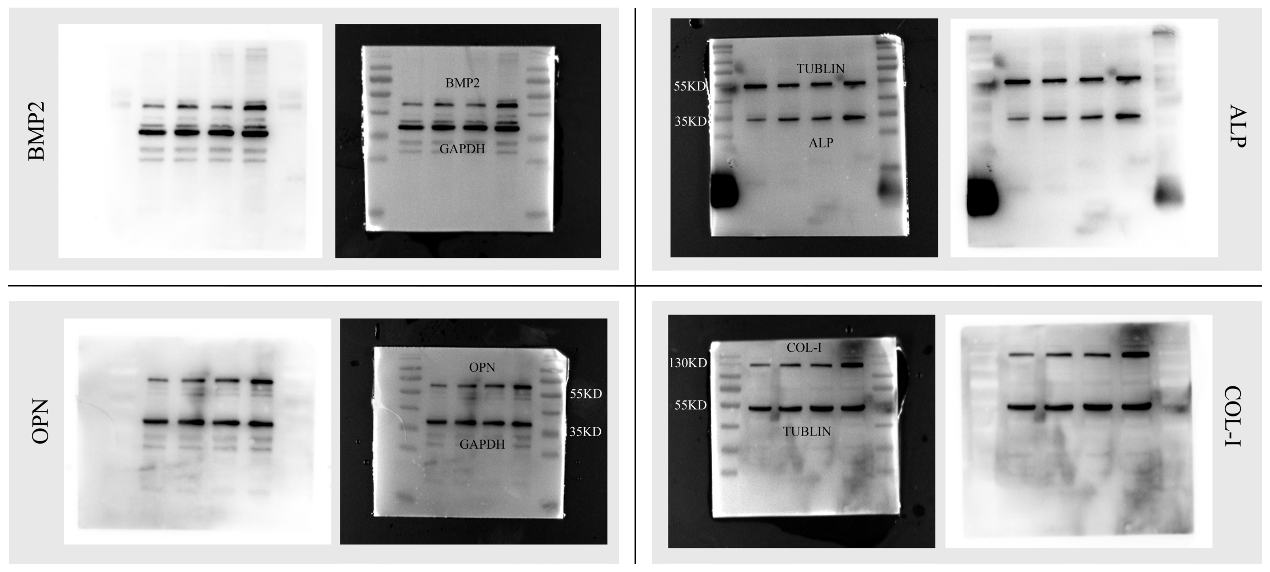


**Figure S7.** Raw data of western-blot analysis in Figure 7b.


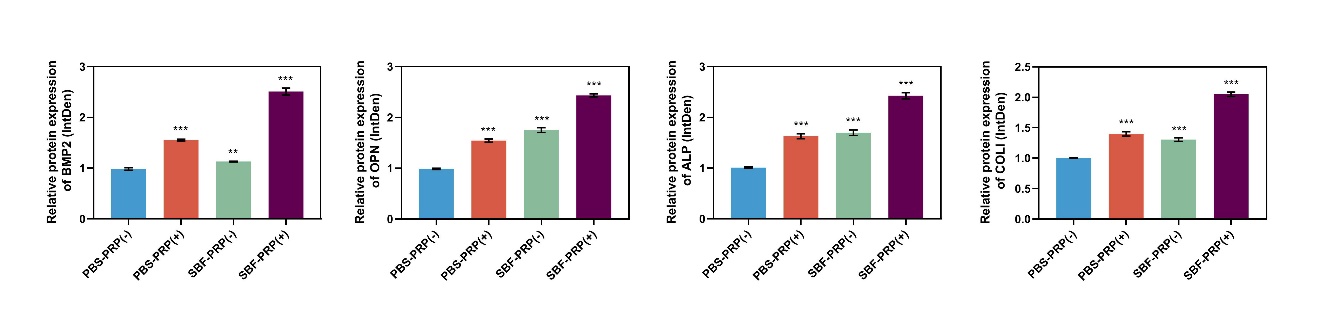


**Figure S8.** Semi quantitative analysis of western blot in Figure 7b.

**Table S1.** Semi-quantitative determination of elemental composition based on EDX spectra

**
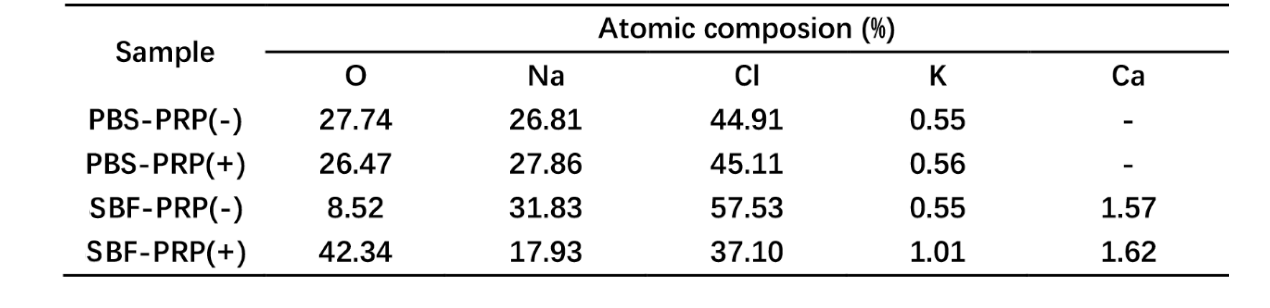
**

**Table S2.** Sequences of PCR primers used in this study


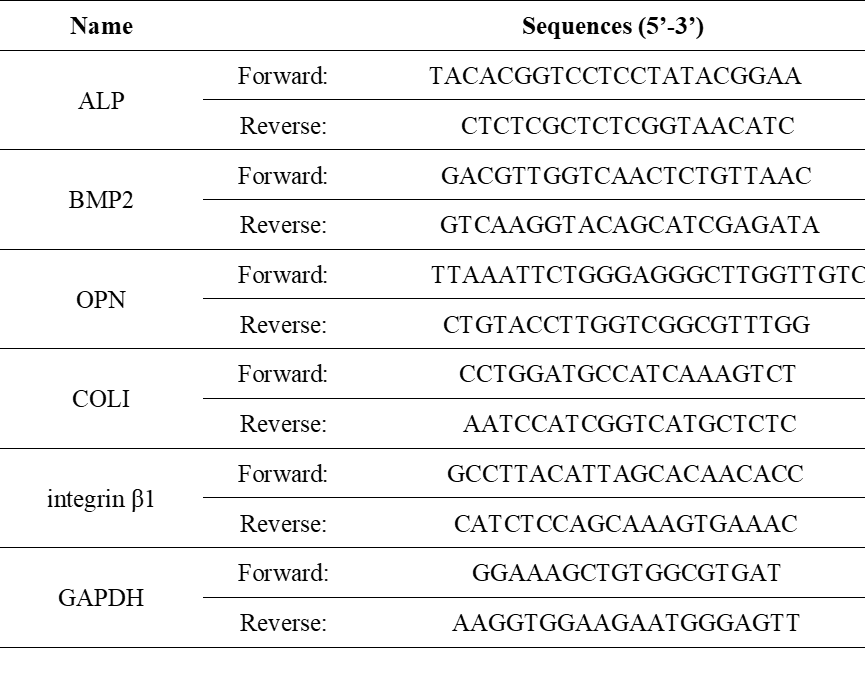


**Table S3.** Numerical results Cell viability of BMSCs after cultivated on four groups of hydrogels for 1 and 3 days respectively

|  | **PBS-PRP(-)** | **PBS-PRP(+)** | **SBF-PRP(-)** | **SBF-PRP(+)** |
| --- | --- | --- | --- | --- |
| **Day 1** | 0.998±0.012 | 1.030±0.008 | 0.999±0.009 | 1.007±0.043 |
| **Day 3** | 1.007±0.011 | 1.027±0.046 | 0.9647±0.022 | 0.988±0.009 |
